# BAC-Pool Sequencing and Assembly of 19 Mb of the Complex Sugarcane Genome

**DOI:** 10.3389/fpls.2016.00342

**Published:** 2016-03-23

**Authors:** Vagner Katsumi Okura, Rafael S. C. de Souza, Susely F. de Siqueira Tada, Paulo Arruda

**Affiliations:** ^1^Centro de Biologia Molecular e Engenharia Genética, Universidade Estadual de CampinasCampinas, Brazil; ^2^Laboratório Central de Tecnologias de Alto Desempenho em Ciências da Vida, Universidade Estadual de CampinasCampinas, Brazil; ^3^Departamento de Genética e Evolução, Instituto de Biologia, Universidade Estadual de CampinasCampinas, Brazil

**Keywords:** sugarcane, BAC pool sequencing, synteny, sorghum, sugarcane genome

## Abstract

Sequencing plant genomes are often challenging because of their complex architecture and high content of repetitive sequences. Sugarcane has one of the most complex genomes. It is highly polyploid, preserves intact homeologous chromosomes from its parental species and contains >55% repetitive sequences. Although bacterial artificial chromosome (BAC) libraries have emerged as an alternative for accessing the sugarcane genome, sequencing individual clones is laborious and expensive. Here, we present a strategy for sequencing and assembly reads produced from the DNA of pooled BAC clones. A set of 178 BAC clones, randomly sampled from the SP80-3280 sugarcane BAC library, was pooled and sequenced using the Illumina HiSeq2000 and PacBio platforms. A hybrid assembly strategy was used to generate 2,451 scaffolds comprising 19.2 MB of assembled genome sequence. Scaffolds of ≥20 Kb corresponded to 80% of the assembled sequences, and the full sequences of forty BACs were recovered in one or two contigs. Alignment of the BAC scaffolds with the chromosome sequences of sorghum showed a high degree of collinearity and gene order. The alignment of the BAC scaffolds to the 10 sorghum chromosomes suggests that the genome of the SP80-3280 sugarcane variety is ∼19% contracted in relation to the sorghum genome. In conclusion, our data show that sequencing pools composed of high numbers of BAC clones may help to construct a reference scaffold map of the sugarcane genome.

## Introduction

Grasses have evolved by the complete duplication of their chromosome sets. Some grass species show variable degrees of ploidy and high content of repetitive sequences (Wang et al., 2010, 2011). This is true in the case of the sugarcane genome. The modern sugarcane varieties are hybrids derived from crosses between *Saccharum officinarum*, which has a chromosome constitution of 2*n* = 80, and *S. spontaneum*, which has a chromosome constitution of 2*n* = 40–128 (Cheavegatti-Gianotto et al., 2011). The commercial varieties grown worldwide have been selected from the populations produced by a few backcross cycles between the interspecific hybrid and the high sugar content parent *S. officinarum*. This crossing and selection scheme resulted in varieties with chromosome constitutions varying between 2*n* = 100–130 with 5–20% of the chromosomes inherited from *S. spontaneum*, 70–80% inherited from *S. officinarum* and recombinant chromosomes formed from homeologous chromosomes of both species (Grivet and Arruda, 2001). This complex genomic architecture with multiple homo/homeoalleles at each locus (Daugrois et al., 1996; Guimaraes et al., 1999) makes assembling very difficult using shotgun sequencing, as reads arising from homeoalleles would collapse, making it difficult to recover large consensus sequences or contigs. As a consequence, the complete sequence of the sugarcane genome has not yet been assembled, and it could be envisaged that to some extent a sugarcane consensus genome sequence may comprise mosaic sequence arrangements with impaired biological meaning. However, partial alignment of overlapping regions of large contigs would help understand the genome organization, as different homologous/homeologous chromosome segments would be represented in the alignments. Such a reference map could be created by sequencing bacterial artificial chromosome (BAC) libraries and aligning the sequences using the sorghum genome sequence as a syntenic template (Paterson et al., 2009).

Efforts to sequence sugarcane BAC clones have been reported previously (de Setta et al., 2014). In this case, the sequences were generated by individually sequencing and assembling each BAC clone. However, this strategy is time consuming and costly because sequencing libraries must be generated from the DNA individually isolated from each BAC clone. An alternative is sequencing pools of BAC clones, preferably without previous mapping, covering the entire genome. BAC pool sequencing has been used to generate megabases (MB) of genome sequence for several species. For example, 3 Mb of rice sequences were generated from six pools composed of 28 BAC clones, each using the 454 sequencing platform (Rounsley et al., 2009). In another example, a pool composed of eight BACs was used to generate 1 Mb of sequences from the salmon genome using the 454 platform (Quinn et al., 2008). In these two cases, the number of BACs per pool was very small, and the authors used the minimum tiling path to fingerprint the pooled BACs. In a third example, two pools of 35 and 23 BACs from a BAC library constructed from a melon line were sequenced using the 454 platform (González et al., 2010).

In this report, we describe the sequencing of a sugarcane BAC pool composed of a large number of BACs as a cost-effective way of generating large contigs of non-overlapping BAC clones. By randomly sampling BAC clones from a sugarcane BAC library (Figueira et al., 2012), we were able to generate 19.2 Mb of sequences assembled into 2,451 scaffolds with a minimum sequence size of 2 Kb. By syntenic alignment of scaffolds to the sorghum genome, we could assess scaffold completeness, the randomness distribution of the scaffolds along the sorghum chromosomes, the sugarcane/sorghum synteny and the gene and repetitive sequence content of a sample of the sugarcane genome.

## Materials and Methods

### BAC Library

The SS_SBa BAC library comprises 36,864 clones prepared with genomic DNA isolated from the sugarcane variety SP80-3280, by partial digestion with HindIII and ligation into the pAGIBAC1 vector (Figueira et al., 2012). The library represents approximately six genomic equivalents of the monoploid sugarcane genome.

### BAC Library Size Determination and Pooling

A total of 192 BAC clones were randomly selected from the 96 × 384-well plates, two for each plate, and re-plated into two 96-well plates. Clones were grown overnight, and the cultures were used to prepare three additional replicates for the two 96-well plates that were stored at -80°C in Circle Grow medium containing 20% glycerol. The sizes of the clone inserts were estimated using NotI restriction enzyme digestion (Figueira et al., 2012). *Escherichia coli* harboring each one of the 192 clones were individually grown overnight in 50 ml falcon tubes containing 10 mL of Circle Grow medium and 12.5 μg/mL chloramphenicol at 37°C and 300 rpm. A total of 178 clone cultures with growth at ODs ranging from 0.6 to 1.0 (Supplementary Table S1) were pooled, pelleted and the DNA extracted using the QIAGEN Large-Construct Kit.

### Illumina Sequencing

One microgram of DNA prepared from the BAC pool was used to prepare small-insert libraries (150, 400, and 800 bp). For this, the DNA was randomly fragmented by sonication using Bioruptor (Diagenode, Denville, NJ, USA) and the desired fragments were size-selected by gel electrophoresis. Illumina paired-end sequencing libraries were prepared using the Truseq DNA sample preparation Kit V2 and sequenced on a HiSeq2000 platform. Sonication, library preparation and sequencing were performed at the Central Laboratory of High Performance Technologies (LaCTAD) of the Universidade Estadual de Campinas^1^.

### PacBio Sequencing

A total of 23 μg of BAC pool DNA was submitted to the Duke University Genome Sequencing and Analysis Core Resource^2^ for sequencing using the PacBio platform. One large insert library (4–10 kb) was sequenced in one SMRT cell using the XL-C2 chemistry.

### Sequence Assembly

The Illumina reads were pre-filtered using quality criteria (90% of bases with phred quality ≥30) and primer/adaptor contamination removal using the NGS QC Toolkit (Patel and Jain, 2012). Reads of vector pBeloBAC11 and *E. coli* DH10B (CP000948) genomic DNA were identified using Bowtie (Langmead et al., 2009) and removed by custom Perl scripts. Assembly of the Illumina reads was performed using Edena (Hernandez et al., 2008). The PacBio sequence data were uploaded to the SMRT Analysis Software v2.1.1^3^, and by applying RS_CeleraAssembler protocol, reads were error corrected with 400X coverage Illumina reads using PacBioToCa (Koren et al., 2012). The corrected reads were assembled with Celera Assembler (Myers et al., 2000; PacBio contigs). The Illumina and PacBio contigs were assembled with the Celera Assembler (wgs8.0). Hybrid scaffolding of the Illumina contigs using PacBio reads was performed using SSPACE-LongRead (Boetzer and Pirovano, 2014) and (A Hybrid Assembler; Bashir et al., 2012), a module of the SMRT Analysis Software. In addition to standard assembly metrics (number of contigs/scaffolds, largest sequence length, N50), sugarcane BAC end sequences (BESs; Figueira et al., 2012) positioning in the assembled contigs/scaffolds was used to validate assemblies. The number of BESs uniquely anchored at the end of a contig/scaffold (less than 1000 nucleotides from the sequence end) was considered a parameter to verify the consistency of an assembly (number of correctly anchored BESs). BES positions in the contigs/scaffolds were determined using BlastN (*e*-value cutoff of 1e^-10^). BESs uniquely positioned at the middle of contigs/scaffolds contributes negatively to the assembly. A complete BAC sequence (“One Contig”) was determined as the contig/scaffold that had its corresponding BES pair mapped at the end of its sequence and had a length similar to the expected BAC length.

### Sequence Analysis

Repeat element identification and masking were performed using the Censor (Kohany et al., 2006) software using grass sequences from Repbase (Jurka et al., 2005). The repeat masked versions of the scaffold sequences were submitted to gene prediction processing. Genes were predicted using the EVidenceModeler (EVM; Haas et al., 2008) annotation tool by combining predictions from Augustus (Stanke et al., 2008), GlimmerHMM (Majoros et al., 2004), and GeneMark (Lomsadze et al., 2005). EST alignments were processed by PASA (Haas et al., 2003) using SUCEST EST sequences (Vettore et al., 2003). Predicted genes were searched against Swissprot, Uniref90, and the NCBI non-redundant protein database using BlastX (*e*-value cutoff of 1e^-5^) and searched against SUCEST EST and sorghum CDS using BlastN (*e*-value cutoff of 1e^-10^). The Blast2GO software was used to determine GO terms and protein codes. Masked scaffold sequences ≥2,000 bp were mapped to the sorghum chromosomes using BlastN (*e*-value cutoff of 1e^-10^) and Perl and shell scripts. High-scoring segment pairs (HSP) were sorted by scaffold positioning, and an ‘expanded alignment’ was determined by joining non-overlapping HSPs. Scaffolds with a minimum of 1,000 bp expanded alignment length were considered mapped to the sorghum chromosomes. Synteny analysis between sugarcane and sorghum was performed based on the expanded alignment.

## Results

### Sequencing and Assembling of BAC Pool DNA

The viability of our strategy to sequence and assembly pools of BACs were tested using a random sample of BACs from the BAC library of sugarcane SP80-3280 containing ∼37,000 clones (Figueira et al., 2012). A total of 178 clones were successfully grown and used for DNA extraction. An equimolar amount of DNA from each BAC were pooled and used for Illumina and PacBio sequencing library preparation. For sequencing on the Illumina platform, we prepared paired end libraries with insert sizes of 170, 400, and 800 bp using the DNA pool from the 178 BAC clones. Libraries were sequenced in a single lane of the HiSeq2000 resulting in 24.6 Gb of usable reads (Supplementary Table S2). The size of each BAC clone used to construct the pool was previously estimated by NotI restriction endonuclease analysis (Figueira et al., 2012; Supplementary Table S1). The sum of the sizes of the 178 BACs was estimated to be 21.7 Mb. Thus, the sequence reads produced by the HiSeq2000 platform were in excess of 1,000-fold coverage of the estimated sum of the BAC clone sequences. The same DNA pool was sequenced using the PacBio SMRT sequencing platform. Using a single Smart Cell, we produced 101,841 reads with an average length of 3,637 bp totaling 370.4 Mb of sequence corresponding to 17-fold coverage of the estimated sum of the BAC clone sequences (Supplementary Table S3).

We tested three hybrid-assembling strategies to assemble the BAC pool sequence reads produced by the two sequencing platforms (Supplementary Figure S1). The PacBio sequencing platform produces long sequence reads, but these reads possess 15–20% base errors while the Illumina sequencing platform produces shorter sequence reads but with higher base accuracy. Therefore, in the first strategy, the Illumina reads were used for error correction of the PacBio long reads and then the corrected long PacBio reads were assembled using the Celera Assembler (Myers et al., 2000). In the second strategy, the Illumina reads were first assembled using Edena (Hernandez et al., 2008), and then a hybrid assembly was performed using the Illumina assembled contigs and the PacBio contigs assembled in the first strategy. The hybrid assembly was performed using the Celera Assembler. In the third strategy, hybrid scaffolding was performed in which the PacBio corrected reads were used to anchor the Illumina assembled contigs. The assembly results from the three different strategies were examined according to standard assembly metrics (number of contigs/scaffolds, largest contig/scaffold length, N50 value) and two additional criteria: anchoring of BAC end sequences (BESs; Figueira et al., 2012) to the assembled scaffolds and the number of large contigs corresponding to the estimated BAC size (Supplementary Table S4). Scaffolds generated by the hybrid assembler (AHA; Bashir et al., 2012) tool produced the best assembly, which was chosen as the reference assembly of the BAC pool sequences. While not having the best N50 value, AHA assembly resulted in a lower number of scaffolds, scaffolds with the largest sizes, the largest number of BESs correctly anchored at the scaffold ends and the highest number of contigs corresponding to complete BAC sequences. The AHA assembly generated 2,451 scaffolds corresponding to a total of 19.2 Mb sequences (**Table 1**), which accounted for 88.2% of the 21.7 Mb estimated sum of bases of the 178 BACs in the pool. The difference between the 19.2 Mb assembled sequences, and the 21.7 Mb estimated sum of BAC sizes could be due the inaccuracy of BAC size estimation using partial restriction digestion and gel electrophoresis fractionation.

**Table 1:** Size distribution of scaffolds assembled using A hybrid assembly (AHA) strategy.

Scaffold length	N° scaffolds	Total bases	Bases (% total)
<2,000	1,758	743,310	3.88
2,000–10,000	321	1,700,748	8.88
10,000–20,000	104	1,480,189	7.73
20,000–40,000	110	3,309,119	17.27
40,000–60,000	63	3,087,796	16.12
60,000–80,000	36	2,464,222	12.86
80,000–100,000	31	2,715,881	14.18
100,000–120,000	7	797,899	4.17
120,000–140,000	16	2,062,825	10.77
140,000–160,000	4	590,630	3.08
>160,000	1	203,132	1.06

Total	2,451	19,155,751	

Estimated total bases	21,717,887	

Scaffolds larger than 20 Kb accounted for ∼80% of the assembled sequences. A total of eight BACs were recovered as one contig compared with the estimated BAC size. The one contig scaffolds were considered complete assembled BACs as their BES anchored exactly at the termini of the scaffolds (Supplementary Table S5). Furthermore, scaffolds with one unique correctly anchored BES were analyzed and 32 additional BACs represented by two scaffolds have sum equivalent of the estimated BAC size (Supplementary Table S6)

### BAC Assembly Validation

Collinearity of AHA assembled scaffolds with sorghum genome, and public sequences of other sugarcane BACs libraries was used to validate correctness of assembly. Collinearity analysis of the sugarcane scaffolds with the sorghum chromosomes showed 133 scaffolds sharing two or more collinear genes with the sorghum chromosomes indicating the preserved gene order and correctness of the assembly (Supplementary Table S7). The recovery of BAC clones with complete insert sequences along with the syntenic gene orders with the sorghum chromosomes represent additional validation of the correctness of the AHA assembled scaffolds. Finally, we retrieved from NCBI, the nucleotide sequences of two sugarcane BACs (GI:530278086, GI:530279041) that matched to four sugarcane scaffolds assembled from our BAC pool sequencing. The alignments showed a high level of sequence identity indicating the high accuracy of the assembled nucleotide sequences of our scaffolds (Supplementary Figure S2). Thus, we concluded that the sequencing strategies used in this work to generate short high accuracy reads from the Illumina platform and long reads from the PacBio platform and the use of the AHA assembling process resulted in the assembly of highly accurate long contigs of the sugarcane genome from pools of a high number of BAC clones in a cost effective manner.

### Content and Nature of Repetitive Sequences

Sequence analysis of the 19.1 Mb assembled nucleotides revealed a content of 54.6% of repetitive sequences among which transposable elements were the predominant group comprising 53.3% of the total repetitive sequence bases. Among the group of transposable elements, the long terminal repeat (LTR) category was the most abundant comprising 43.3% of the total bases, followed by DNA transposons with 7.7% and non-LTR retrotransposons with 2.25% (**Table 2**). Among the LTR group, the *Gypsy* and *Copia* elements accounted for 30.3 and 12.9%, respectively, of the assembled nucleotides. Simple repeats, integrated viruses, and unclassified repeat sequences accounted for 1.08, 0.23, and 0.02% of the total bases, respectively. These data are in accordance with repetitive elements found previously in a total of 317 sequenced sugarcane BACs (de Setta et al., 2014). We previously estimated a slightly smaller proportion of repetitive regions (45.6%) based on the BAC end sequences (Figueira et al., 2012). Our new estimates are more accurate as they are based on a large sequence dataset. The ratio of *Gypsy* and *Copia* LTR elements was 2.3:1, which is higher than that observed in the 317 sequenced sugarcane BACs (de Setta et al., 2014).

**Table 2:** Summary of repetitive sequences among the sugarcane bacterial artificial chromosome (BACs).

Repeat element	Number of elements	Length (bp)	% of Total bases
Transposable element	1,279	10,209,529	53.30
DNA transposon	407	1,479,344	7.72
EnSpm/CACTA	102	545,515	2.85
Harbinger	77	282,933	1.48
Helitron	28	74,200	0.39
Mariner/Tc1	11	7,401	0.04
MuDR	44	218,853	1.14
hAT	87	162,922	0.85
Other	58	187,520	0.98
LTR retrotransposon	732	8,297,946	43.32
Copia	291	2,473,755	12.91
Gypsy	426	5,795,891	30.26
Other	15	28,300	0.15
Non-LTR retrotransposon	138	431,477	2.25
Other	2	762	0.004
Simple repeat	9	206,466	1.08
Satellite	9	206,466	1.08
Integrated virus	3	43,599	0.23
Caulimoviridae	3	43,599	0.23
Unclassified	5	3,965	0.02

Total of repeat elements	1,296	10,463,559	54.62

Total of assembled bases		19,155,751	100.00

### Syntenic Mapping of Scaffolds to the Sorghum Chromosomes

**FIGURE 1:**
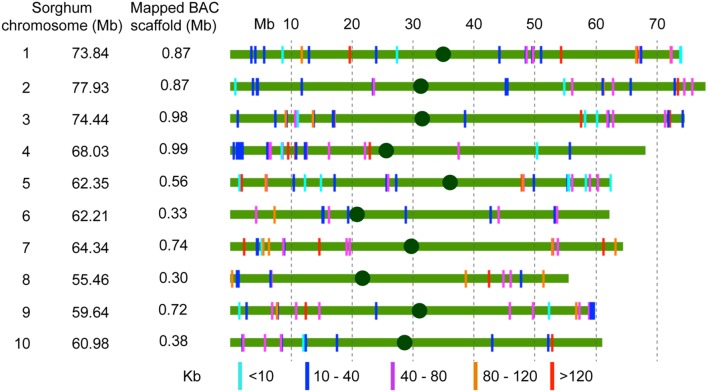
**Orthologous alignment of the assembled BAC scaffolds on the 10 sorghum chromosomes.** Scaffold sequences were aligned along the sorghum chromosome sequences. Repetitive sequences were masked to avoid misalignment. The colored solid lines represent the sorghum chromosomes. The colored vertical bars represent the sugarcane scaffolds.

A total of 292 scaffolds corresponding to 12.4 Mb (67.8% of assembled sequences) with a minimum size of 2 Kb were mapped by syntenic sequence alignment to the nucleotide sequences of sorghum chromosomes (**Table 3**; Supplementary Table S8). The repetitive sequences were masked to avoid misalignment of scaffolds at several locations within and among the sorghum chromosomes. In general, the scaffolds aligned with high accuracy and were homogeneously distributed along the 10 sorghum chromosomes, except for the chromosomes 6, 8, and 10, which had smaller numbers of mapped scaffolds (**Figure 1**). Representation of the scaffolds was slightly higher in chromosomes 1, 3, and 5. No mapped scaffolds corresponded to sequences with high repeat sequence composition. Regarding localization, the 292 scaffolds aligned homogeneously over the sorghum chromosomes (**Figure 1**). This uniformity of alignment must be directly related to the random selection of the BACs clones.

**Table 3:** Scaffolds longer than 2,000 bp mapped to sorghum chromosomes.

Chromosome	Total bases	Bases (% Total)	N° Scaffolds	Scaffold size range
1	1,821,039	9,89	42	3,008–203,132
2	1,607,121	8,73	36	3,106–152,524
3	1,589,753	8,63	44	2,882–138,830
4	1,716,141	9,32	37	3,734–141,339
5	1,121,959	6,09	40	2,086–125,065
6	689,413	3,74	15	6,387–97,701
7	1,268,000	6,89	22	7,190–151,964
8	722,184	3,92	14	10,282–123,118
9	1,270,664	6,90	24	4,562–137,971
10	675,823	3,67	18	2,688–135,690

No Mapped	5,930,344	32,21	401	2,003–129,783

Total	18,412,441		693	

### Gene Content and Distribution among Scaffolds

The annotation pipeline based on *ab initio* gene predictions combined with spliced alignments of transcripts generated a set of 1,338 gene models. Predicted genes were distributed in 431 scaffolds, which correspond to 15.4 Mb (80.57%) of the total assembled sequences (Supplementary Table S9). Among scaffolds containing predicted genes, 245 sequences have two or more genes, and 16 sequences have ten or more genes. Gene density was estimated to be 3.1 genes per scaffold with a coding average size of 713 bp, exon average size of 246 bp and intron average size of 647 bp. A total of 884 genes (66.1%) presented similarity to protein databases, with 565 (63.9%) of them being supported by sugarcane EST sequences (SUCEST; Vettore et al., 2003). Genes were classified using the gene ontology (GO) functional categories (Supplementary Figure S3). A total of 2,330 GO terms were assigned to 558 genes. The Biological Process GO category comprised 41.9% of the identified terms, with the most representative classes being involved in metabolic, cellular, and single-organism processes. Catalytic activity and binding were the two most representative classes in the Molecular Function category (33.4% of terms). Most of the terms were assigned to cell, organelle and membrane classes for the Cellular Component category (24.7% of terms). Collinearity of genes between sugarcane and sorghum was found in 133 scaffolds (≥2 genes) containing 431 genes (Supplementary Table S7).

### Sugarcane and Sorghum Genome Comparison

A customized BLAST pipeline was applied to map the sugarcane scaffolds onto the sorghum chromosomes and determine the syntenic regions. Our results show expanded and contracted regions between sugarcane and sorghum (Supplementary Figure S4). A summary of the expanded and contracted regions shows a positive rate of sorghum syntenic regions in relation to sugarcane on all sorghum chromosomes (1.04–1.41; **Table 4**). Taking all the regions into account, a total of 6,550,682 bp of sugarcane syntenic regions were aligned to 7,809,102 bp of sorghum chromosomes, showing an expansion of the sorghum genome of 19% compared to the sugarcane BAC scaffolds. This result is in accordance with previous studies where the sorghum genome was found to be approximately 20–30% longer than the sugarcane genome (Wang et al., 2010; Figueira et al., 2012).

**Table 4:** Expanded and contracted regions between sorghum chromosomes and sugarcane scaffolds.

Sorghum chromosome	Number of scaffolds	Scaffold mapped size	Chromosome mapped size	Syntenic sorghum/sugarcane rate	Sum of sugarcane expanded regions (bp)	Sum of sugarcane contracted regions (bp)	Sum of sorghum expanded regions (bp)	Sum of sorghum contracted regions (bp)
1	42	866,716	905,270	1.04	556,830	309,886	608,863	296,407
2	36	857,217	1,124,624	1.31	547,673	309,544	830,799	293,825
3	44	977,446	1,036,227	1.06	627,837	349,609	601,346	434,881
4	37	994,271	1,115,670	1.12	708,032	286,239	653,976	461,694
5	40	555,615	721,043	1.30	327,292	228,323	568,417	152,626
6	15	328,515	455,977	1.39	163,097	165,418	354,613	101,364
7	22	705,244	856,835	1.21	418,892	286,352	635,808	221,027
8	14	296,242	417,336	1.41	140,887	155,355	320,335	97,001
9	24	590,229	699,434	1.19	384,530	205,699	449,331	250,103
10	18	379,187	476,686	1.26	281,509	97,678	264,014	212,672

Total	292	6,550,682	7,809,102	1.19	4,156,579	2,394,103	5,287,502	2,521,600

## Discussion

Sequencing the sugarcane genome is challenging due to the interspecific hybrid nature of the crop, the high degree of ploidy and the high proportion of repetitive DNA sequences. Furthermore, the presence of variable sequence size along non-coding and repetitive regions among multiple homologous and homeologous chromosomes makes it difficult to use shotgun approaches from NGS platforms such as Illumina that generate short reads. The strategy to sequence BAC libraries prepared from sugarcane genomic DNA has been suggested to avoid this difficulty (Wang et al., 2010; de Setta et al., 2014). However, sequencing individual BACs is costly and time consuming. In this work, we tested a cost effective strategy to sequence BAC libraries in a pool arrangement. To test this strategy, the Illumina and PacBio platforms were used to sequence a pool of 178 BAC clones randomly sampled from a sugarcane BAC library. Taking the close phylogenetic relationship between sugarcane and sorghum into account, we based our sugarcane BAC pool sequencing rationale on the size of the sorghum genome. The monoploid sorghum genome comprises 10 chromosomes with sizes ranging from ∼55 to 78 Mb (Paterson et al., 2009). The SP80-3280 BAC library used in this work comprises ∼37,000 clones with an average size of ∼120 Kb (Figueira et al., 2012). Thus, we estimated that a pool of ∼200 BACs will account for ∼24 Mb of sequence. If the sugarcane genome has a size similar to the ∼780 Mb sorghum genome, a BAC pool of 200 clones would correspond approximately to ∼3% of the nucleotide sequence of the sugarcane genome. Therefore, we reasoned that if these clones are randomly sampled, there is a 97% chance that they do not overlap. Absence of overlapping would facilitate the assembling process, except for the repetitive sequences, as the reads produced from each of the individual BAC clones in the pool will be recovered in an isolated contig. If this rationale works, it would not be necessary to have any additional information from the individual BACs in the pool.

Bacterial artificial chromosome pooling approach had already been applied before in plants genome assembly (Rounsley et al., 2009; González et al., 2010), with a maximum number of 35 sequenced BACs in 454 platform. Genome assembly via BAC library in a cost effective manner would require a larger number of BACs in the same pool. Illumina sequencing platform offers greater throughput than other technologies and because of that was our main initial choice to sequence BAC pools. However, accurate assembly of complex genomes based solely on short Illumina reads is a still challenging, especially because of high repetitive sequences content and high polyploid architecture of the sugarcane genome. Thus, we decided to incorporate a third-generation sequencing platform (PacBio) that provides longer reads. Hybrid strategies involving second and third generations sequencing technologies have been documented to improve genome assembly of bacteria and other organisms (Bashir et al., 2012), but to our knowledge have never been applied to solve complex polyploidy plant genomes. Our data showed that hybrid assembly is capable of improving assembly metrics when compared with a strategy using only Illumina reads. The assembly was validated by several criteria, and most importantly, the alignment of the scaffolds onto the sorghum chromosomes strongly support the idea that pooling a high number of sugarcane BAC clones randomly chosen from libraries is a very cost effective way to produce a sequence map of the sugarcane genome.

The genome information produced from this work is highly valuable regarding unraveling the structure and sequence composition of the sugarcane genome. This information allows us to conclude, for example, that the sequenced sugarcane scaffolds aligned to sorghum chromosomes are ∼19% contracted in relation to the sorghum syntenic regions. Similar results were presented by (Wang et al., 2010; Figueira et al., 2012), where the sorghum genome was found to be approximately 20–30% longer than the sugarcane genome. This information raises the question whether this is because the assembled sequence represents only 3% of the sugarcane genome or whether this is a particularity of the genome of the SP80-3280 sugarcane variety that may be smaller than the sorghum genome while other BAC sequences produced from the R570 sugarcane variety have indicated a genome size higher than that of the sorghum genome (de Setta et al., 2014).

Even though BAC libraries are particular relevant approach to solve large complex genomes, sequencing individual BAC is costly and laborious. Our approach to sequencing BAC pools is cost effective way to overcome these problems and retrieve important biological information to construct a reference scaffold map of genome. The hybrid assembly with Illumina and PacBio reads provided longer contigs to access aspects of genome architecture, functional traits and synteny. We strongly support usage of this approach to solve complex plant genomes and retrieve a reference map for biological studies.

### Accession

The reads from the BAC pool have been deposited in the NCBI GenBank under the accession PRJNA299804.

## Author Contributions

VO and PA conceived the project; RdeS and SdeST prepared the BAC clone cultures, isolated DNA, prepared the sequencing libraries and executed the Illumina sequencing. VO assembled the BAC sequences and performed the sequence analysis; VO, RdeS and PA wrote the manuscript.

## Conflict of Interest Statement

The authors declare that the research was conducted in the absence of any commercial or financial relationships that could be construed as a potential conflict of interest.

## Footnotes

1 www.lactad.unicamp.br

2 www.genome.duke.edu

3 http://www.pacb.com/devnet
