# Supplementary material for: BAC-Pool Sequencing and Assembly of 19 Mb of the Complex Sugarcane Genome
Source: Front Plant Sci. 2016 Mar 23;7:342. doi: 10.3389/fpls.2016.00342 (PMC4804495; doi:10.3389/fpls.2016.00342)
Supplement: Supplementary file 2 [file Table_2.DOCX]

| **Supplementary Table 2** - Sequencing output Illumina HiSeq2000 sequencing of Sugarcane BAC pool | | | | | | |
| --- | --- | --- | --- | --- | --- | --- |
| Library/  fragmente size | Number of reads | Number of reads after quality filtering | Number of reads after E. coli filtering | Number of reads after pbelo vector filtering | Number of bases after pbelo vector filtering (bp) | Coverage per BAC |
| BAC170 | 123,610,182 | 96,092,842 | 94,999,141 | 86,788,234 | 8,678,823,400 | 400 |
| BAC400 | 173,447,366 | 121,857,784 | 120,665,875 | 108,149,370 | 10,814,937,000 | 498 |
| BAC800 | 123,313,864 | 60,511,394 | 59,824,685 | 51,776,460 | 5,177,646,000 | 238 |
| Total | 420,371,412 | 278,462,020 | 275,489,701 | 246,714,064 | 24,671,406,400 | 1,136 |
