# Supplementary material for: BAC-Pool Sequencing and Assembly of 19 Mb of the Complex Sugarcane Genome
Source: Front Plant Sci. 2016 Mar 23;7:342. doi: 10.3389/fpls.2016.00342 (PMC4804495; doi:10.3389/fpls.2016.00342)
Supplement: Supplementary file 4 [file Table_4.DOCX]

| **Supplementary Table 4** – Comparison of the 3 strategies used for BAC pool sequence assembly | | | | | |
| --- | --- | --- | --- | --- | --- |
| Input | Reads Illumina | Reads Illumina  +  Reads PacBio | Contigs PacBio  +  Contigs Edena | Contigs Edena  + Reads PacBio | Contigs Edena  + Reads PacBio |
| Software | Edena | PacBioToCA  +  Celera Assembler | Celera Assembler | SSPACE-LongRead | AHA |
| Output | Contigs | Contigs | Contigs | Scaffolds | Scaffolds |
| Number of contigs/scaffolds | 3.616 | 3.636 | 2.500 | 2.697 | 2.451 |
| Number of bases in contigs/scaffolds (bp) | 18.667.365 | 19.935.357 | 18.577.208 | 19.085.165 | 19.155.751 |
| N50 (bp) | 16.444 | 11.003 | 28.645 | 56.229 | 54.129 |
| Largest contig/scaffold size (bp) | 125.720 | 137.632 | 146.754 | 195.258 | 203.132 |
| BES matching end of scaffolds |  | 240 | 223 | 255 | 272 |
| BES matching middle of scaffolds |  | 31 | 50 | 51 | 33 |
| BES matching more than one scaffold |  | 35 | 46 | 19 | 20 |
| BES not matching scaffolds |  | 45 | 32 | 26 | 26 |
| Number of “One Contig” scaffolds |  | 0 | 2 | 7 | 8 |
