# Supplementary material for: BAC-Pool Sequencing and Assembly of 19 Mb of the Complex Sugarcane Genome
Source: Front Plant Sci. 2016 Mar 23;7:342. doi: 10.3389/fpls.2016.00342 (PMC4804495; doi:10.3389/fpls.2016.00342)
Supplement: Supplementary file 5 [file Table_5.DOCX]

| **Supplementary Table 5** - BACs assembled in “One Contig” – BES match consistently end of scaffolds and scaffold length is similar to expected BAC length | | | | | | | | | |
| --- | --- | --- | --- | --- | --- | --- | --- | --- | --- |
| **BAC** | **Expected BAC length** | **Scaffold length** | **End Distance BES (.b)** | **End Distance BES (.g)** | **Alignment Coverage**  **Percentage BES (.b)** | **Alignment Coverage Percentage BES (.g)** | **Number of Scaffolds matching BES (.b)** | **Number of Scaffolds matching BES (.g)** | **Scaffold ID** |
| **SCSBa0008C24** | 141,860 | 138,830 | 91 | 96 | 100.00 | 85.58 | 2 | 1 | scaffold5/869 |
| **SCSBa0042A24** | 127,200 | 129,783 | 103 | 1 | 98.89 | 93.96 | 1 | 1 | scaffold5/2173 |
| **SCSBa0045C24** | 142,900 | 126,859 | 93 | 95 | 100.00 | 100.00 | 1 | 1 | scaffold5/770 |
| **SCSBa0055C24** | 112,430 | 141,339 | 119 | 95 | 97.28 | 100.00 | 1 | 1 | scaffold5/2050 |
| **SCSBa0067A24** | 87,380 | 64,808 | 97 | 95 | 93.56 | 100.00 | 1 | 1 | scaffold5/1460 |
| **SCSBa0084A24** | 123,332 | 119,761 | 93 | 97 | 99.46 | 100.00 | 1 | 1 | scaffold5/1840 |
| **SCSBa0085C24** | 114,000 | 123,118 | 97 | 96 | 99.44 | 100.00 | 1 | 1 | scaffold5/2075 |
| **SCSBa0093C24** | 28,995 | 26,337 | 96 | 97 | 100.00 | 99.88 | 1 | 1 | scaffold5/1329 |
