# Supplementary material for: BAC-Pool Sequencing and Assembly of 19 Mb of the Complex Sugarcane Genome
Source: Front Plant Sci. 2016 Mar 23;7:342. doi: 10.3389/fpls.2016.00342 (PMC4804495; doi:10.3389/fpls.2016.00342)
Supplement: Supplementary file 9 [file Table_9.DOCX]

| **Supplementary Table 9:** Distribution of gene models among scaffolds | | |
| --- | --- | --- |
| **Number of scaffolds** | **Number of Gene Models** | **Scaffold Size Range** |
| 1 | 17 | 144,803 - 144,803 |
| 1 | 15 | 122,587 - 122,587 |
| 2 | 14 | 135,690 - 203,132 |
| 1 | 13 | 120,035 - 120,035 |
| 1 | 12 | 151,964 - 151,964 |
| 4 | 11 | 51,028 - 134,888 |
| 6 | 10 | 50,115 - 141,339 |
| 9 | 9 | 49,010 - 131,796 |
| 11 | 8 | 39,928 - 138,830 |
| 24 | 7 | 37,498 - 152,524 |
| 22 | 6 | 39,569 - 127,716 |
| 24 | 5 | 12,278 - 128,730 |
| 28 | 4 | 16,384 - 90,365 |
| 40 | 3 | 5,459 - 80,959 |
| 71 | 2 | 1,695 - 119,761 |
| 186 | 1 | 233 - 129,783 |
| **Total**  431 | 1,338 | **Total Bases:** 15,435,186 |
