# Supplementary material for: BAC-Pool Sequencing and Assembly of 19 Mb of the Complex Sugarcane Genome
Source: Front Plant Sci. 2016 Mar 23;7:342. doi: 10.3389/fpls.2016.00342 (PMC4804495; doi:10.3389/fpls.2016.00342)
Supplement: Supplementary file 10 [file Data_Sheet_1.DOCX]

**Supplementary Figure 1** - Flowchart describing the three strategies for hybrid assembly involving Illumina and PacBio sequence data.


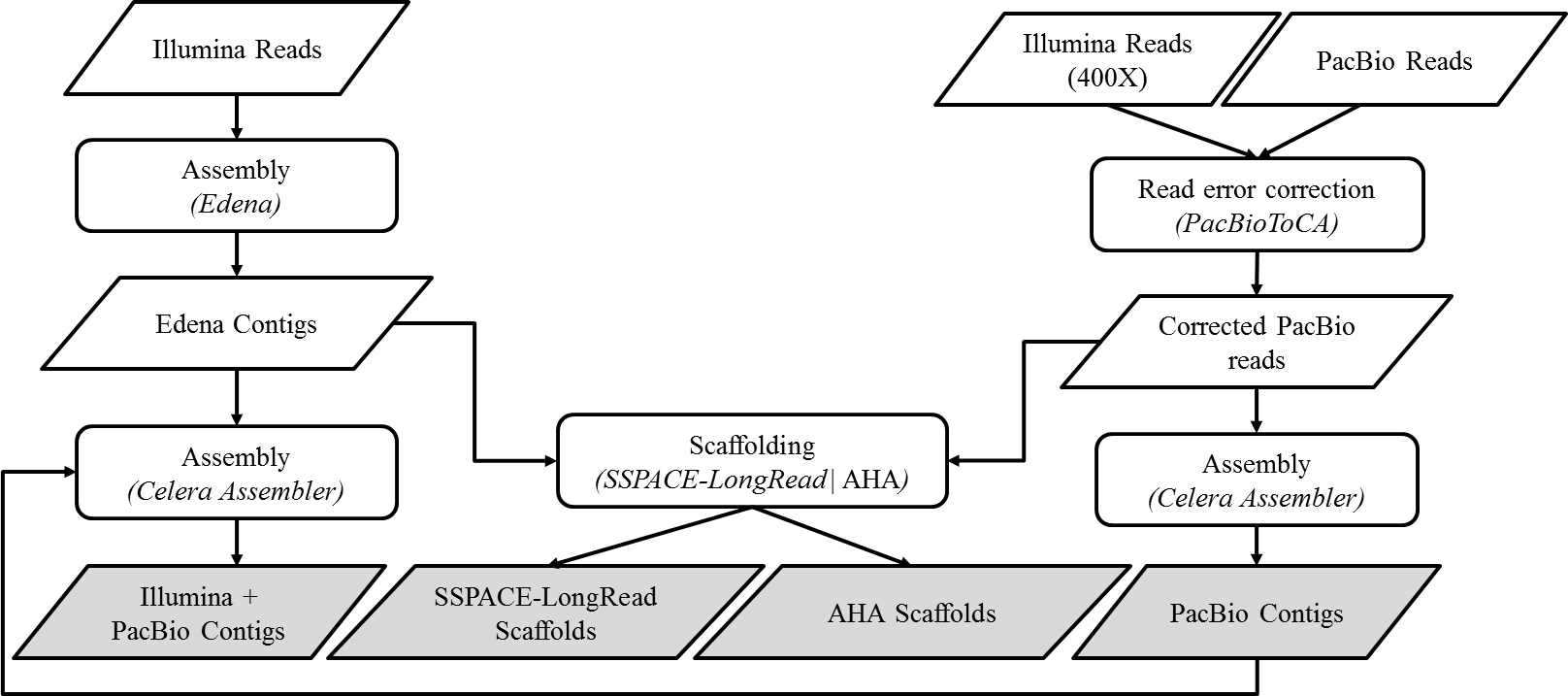


**Supplementary Figure 2-** Alignment of assembled scaffolds nucleotide sequences to nucleotide sequence regions of sequenced BACs deposited in NCBI. In the two cases the scaffolds aligned to contiguous regions of the BACs indicating the correctness of the assemblage.


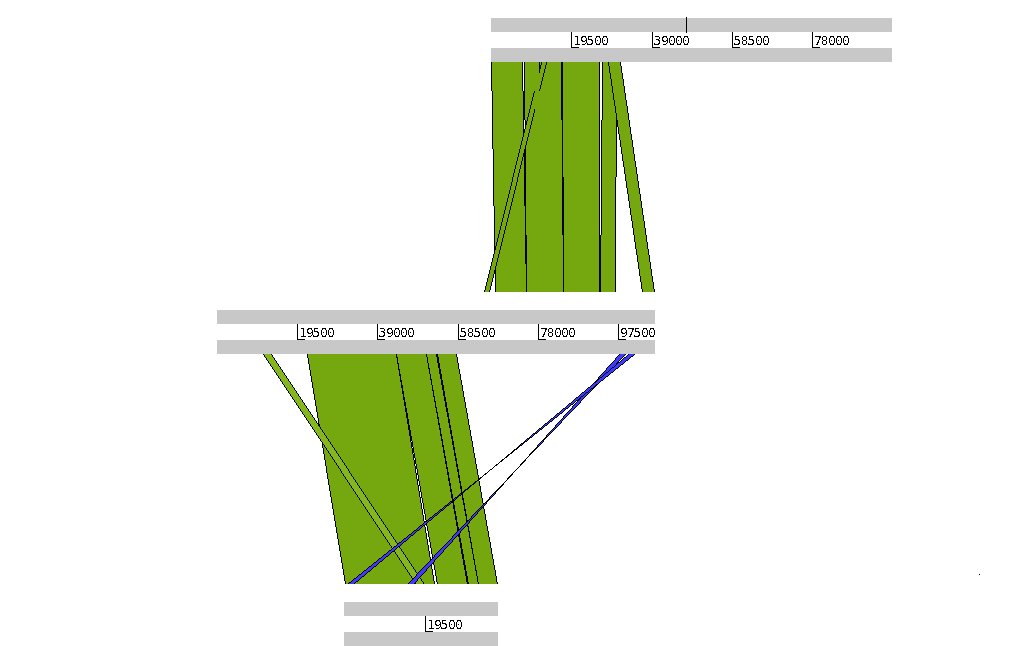


Sugarcane scaffold 1273


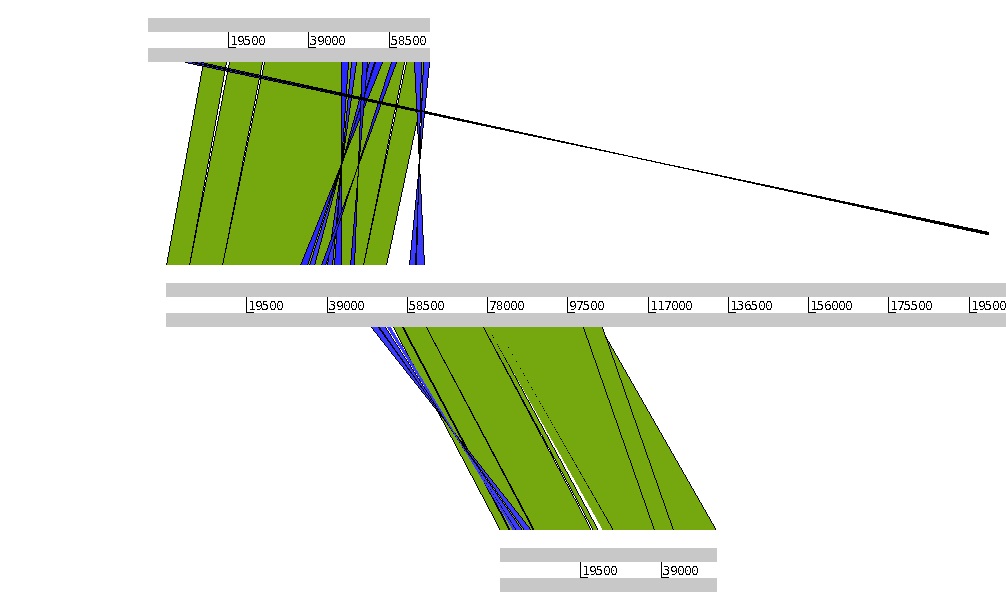


Sugarcane Scaffold 2391

Sugarcane scaffold 1646

Sugarcane BACKF184889.1 – GI:530279041

Sugarcane Scaffold 1582

Sugarcane BAC

KF184940.1 - GI:530278086

**Supplementary Figure 3** - Gene Ontology categorization of sugarcane genes annotated in the 19.2MB of sequences of sequenced scaffolds

**Supplementary Figure 4** - Examples of contracted (A and B) and expanded (C and D) regions of sugarcane scaffolds in relation to overlapping regions of sorghum chromosomes.

**(A)**

Sugarcane Scaffold.1002

Sorghum chromosome 4 (8,406,000-8,517,000)


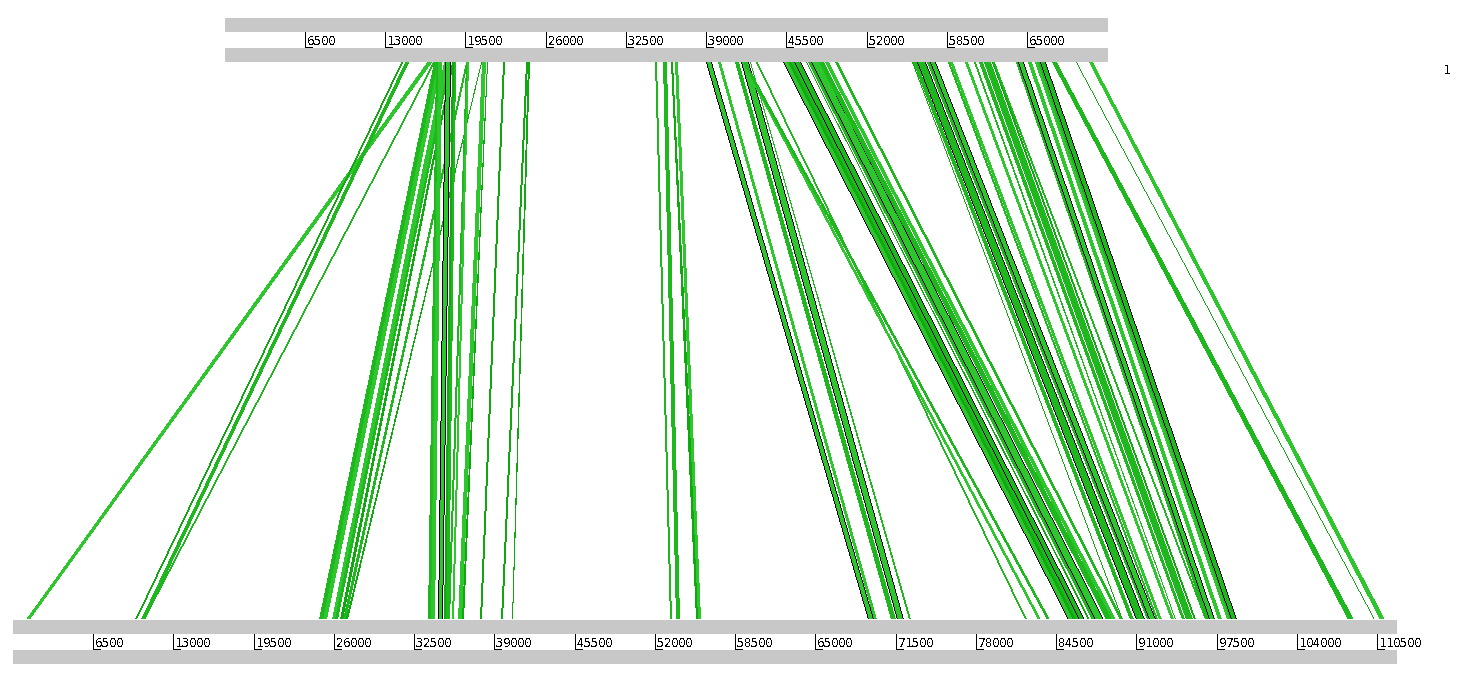


**(B)**


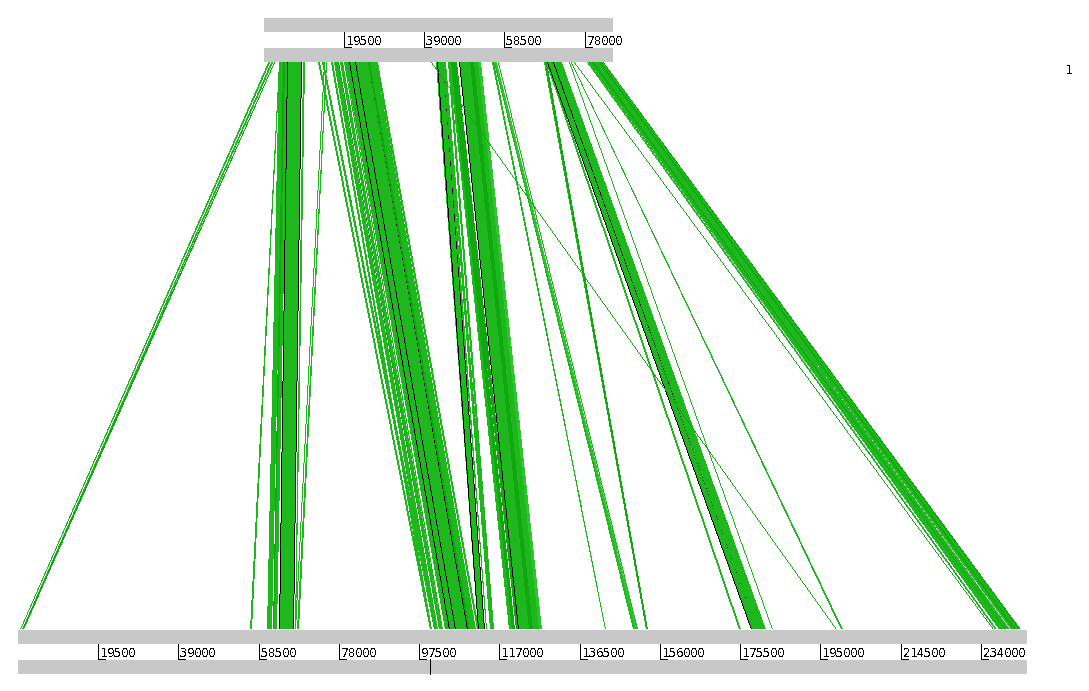


Sorghum chromosome 6 (4,116,000-4,360,000)

Sugarcane Scaffold.1275

**
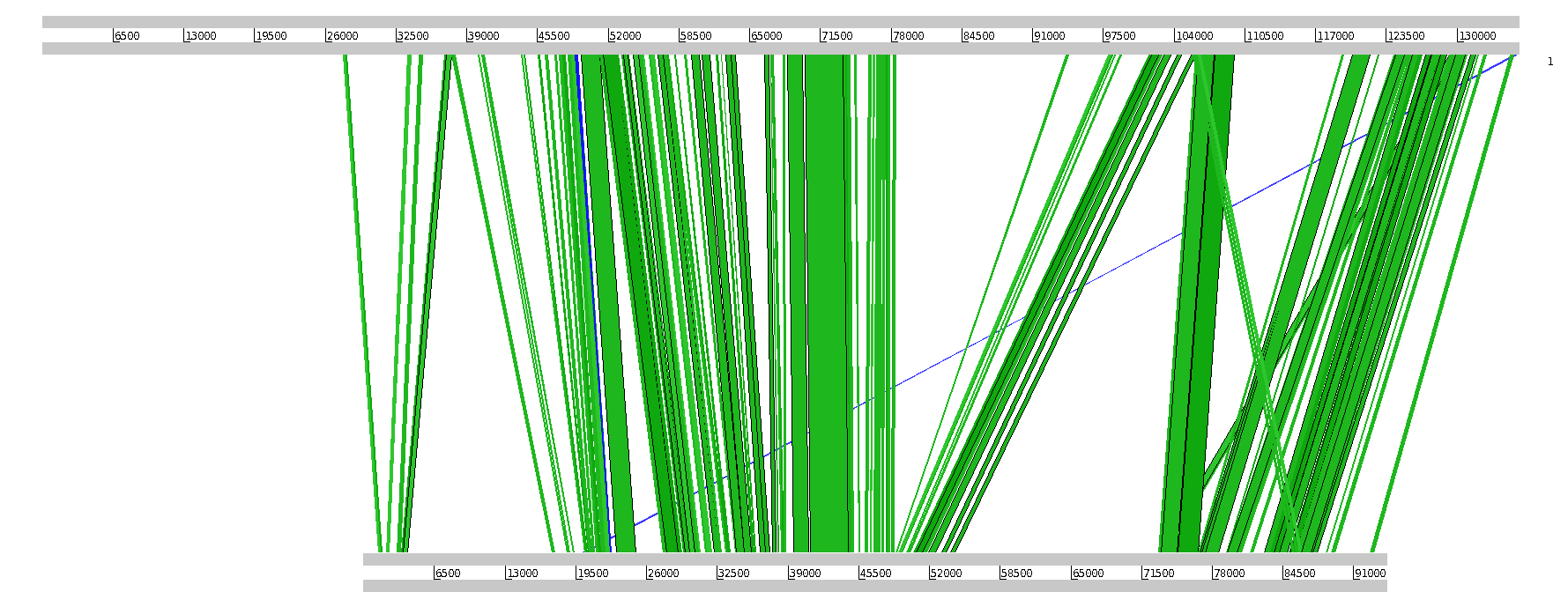
(C)**

Sugarcane Scaffold.1071

Sorghum chromosome 10 (52,659,000-52,750,000)

**
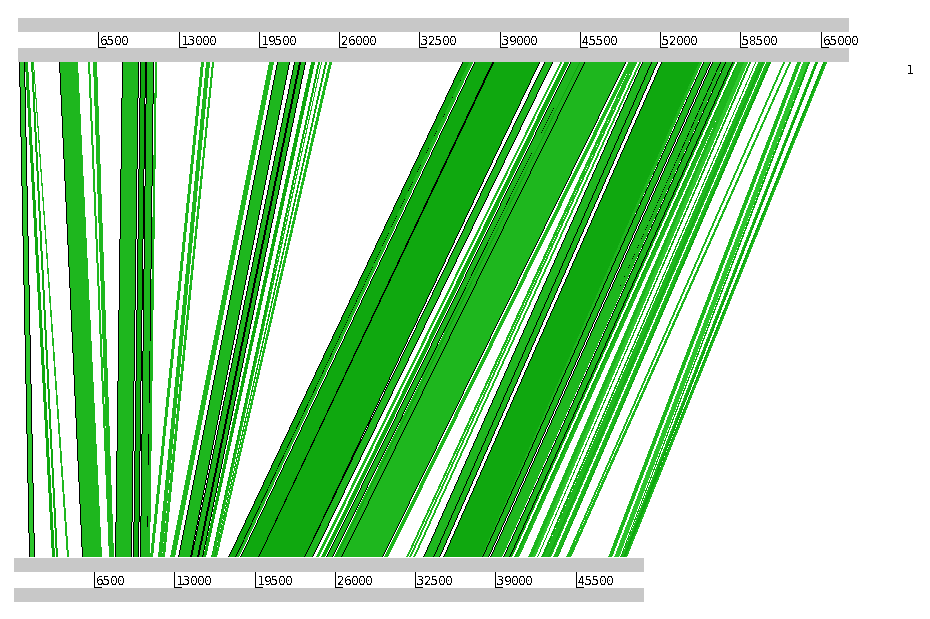
(D**)

Sugarcane Scaffold.1229

Sorghum chromosome 2 (62,725,000-62,774,000)
